# The impact of bipedal mechanical loading history on longitudinal long bone growth

**DOI:** 10.1371/journal.pone.0211692

**Published:** 2019-02-07

**Authors:** Adam D. Foster

**Affiliations:** Department of Anatomy, School of Osteopathic Medicine, Campbell University, Buies Creek, North Carolina, United States of America; Virginia Tech, UNITED STATES

## Abstract

Longitudinal bone growth is accomplished through a process where proliferating chondrocytes produce cartilage in the growth plate, which ultimately ossifies. Environmental influences, like mechanical loading, can moderate the growth of this cartilage, which can alter bone length. However, little is known about how specific behaviors like bipedalism, which is characterized by a shift in body mass (mechanical load), to the lower limbs, may impact bone growth. This study uses an experimental approach to induce bipedal behaviors in a rodent model (*Rattus norvegicus*) over a 12-week period using a treadmill-mounted harness system to test how rat hindlimbs respond to the following loading conditions: 1) fully loaded bipedal walking, 2) partially loaded bipedal walking, 3) standing, 4) quadrupedal walking, and 5) no exercise control. These experimental conditions test whether mechanical loading from 1) locomotor or postural behaviors, and 2) a change in the magnitude of load can moderate longitudinal bone growth in the femur and tibia, relative to controls. The results demonstrate that fully loaded bipedal walking and bipedal standing groups showed significant differences in the percentage change in length for the tibia and femur. When comparing the change from baseline, which control for body mass, all bipedal groups showed significant differences in tibia length compared to control groups. However, there were no absolute differences in bone length, which suggests that mechanical loads from bipedal behaviors may instead be moderating changes in growth velocity. Implications for the relationship between bipedal behaviors and longitudinal bone growth are discussed.

## Introduction

Longitudinal bone growth results from a process where proliferating chondrocytes produce hypertrophic chondrocytes that are aligned with the long axis of the bone. Growth velocity (length/time) is primarily driven by the rate of production of hypertrophic chondrocytes. Proliferative processes that occur between primary and secondary ossification centers (also referred to as the growth plate) form the epiphyses of bones and are responsible for long bone growth throughout adolescence [1]. Cartilaginous regions that make up the diaphysis and epiphysis of the bone ossify over time at a rate that is closely linked with phylogeny [2]. However, there are a number of intrinsic and environmental factors that can modify longitudinal bone growth that are important for explaining intraspecific variation in bone length.

Experimental work exploring intraspecific variation in longitudinal bone growth suggests that differences in bone length can be attributed to processes at the level of the growth plate relating to the timing of cellular events, the initial pool and size of chondrocytes, and the rate of chondrocyte proliferation [3]. One important finding from Rolian [3] is that the initial pool of proliferating chondrocytes at birth explains the majority of variation in bone length in intraspecific comparisons. However, we also know that epigenetic processes like mechanical loading can moderate some of these same variables, which can affect growth. Indeed, experimental data suggest that mechanical loading applied to bone epiphyses can produce longer skeletal elements and a concomitant increase in bone mineral density and content when compared to controls (e.g.,[4–10]). However, the location, type, and direction of loading is particularly important. For example, compressive loads are associated with suppression of longitudinal growth, which is proportional to the load magnitude [4,5,11]. In contrast, tensile loading is correlated with increases in the dimensions of the zones of proliferation and hypertrophy, as well as the number of chondrocytes in the growth plate, which are positively correlated with bone length [4–10]. Therefore, previous work suggests that the magnitude and frequency of loading are important factors for modulating growth plate variables, and ultimately, longitudinal growth [12].

Bone growth can also be modulated by mechanical loads that result from differences in activity level or behavior (e.g., treadmill exercise or jumping). However, these loads combine a variety of strain types (tension, compression, shear, etc.) and are more difficult to characterize. Indeed, experimental studies which explore how behavioral differences impact longitudinal growth (as opposed to applying loads from an external device, like those discussed above), have conflicting results. Some studies show increases in bone length from treadmill exercise and jumping behaviors (e.g.,[9,13]), while others show suppression of growth [14,15]. These differences in outcome may be explained in part by variation in the magnitude, frequency and type of loads applied across individuals in the experiment [12]. We also still know very little about how mechanical loads that are transiently compressive (as opposed to sustained periods of compression), in shear, or in torsion, impact longitudinal growth [8]. One way to address this gap in our knowledge is to develop an experimental method that moves beyond an exercised vs. non-exercised comparison that can impose a greater degree of uniformity in the desired behavior and the amount of mechanical loading that is applied to bone.

Bipedalism offers a unique opportunity to test how mechanical loading from a specific behavior may impact longitudinal bone growth. In bipeds, the pelvic limbs support nearly all body mass, as opposed to sharing the load with the pectoral limbs like in quadrupeds. If the magnitude of mechanical loading is a factor in moderating longitudinal bone growth, it follows that if bipedal loads are placed on the hindlimbs of a non-obligate biped, the longitudinal growth of the femur and tibia may be modified. Previous work suggests that bipedal mechanical loads could play some role in explaining variation in bone growth in humans. For example, limb length is correlated with age during growth and development, but the predictive power of this relationship is low at very young ages [16]. This points to the possibility that variation in limb length in children may be influenced by the age of onset of bipedal walking, and thus alterations in the magnitude of mechanical loads. Indeed, although not directly related to length, there is also a correlation between measures of bone strength in older adults and a late age of onset of walking [17]. Previous work has also found a link between relatively higher mediolateral ground reaction forces in immature gaits and both femoral shape and trabecular orientation [18,19]. These findings provide evidence that mechanical loads from bipedal behaviors may explain some variation in bone growth and phenotype.

One way to test the relationship between bipedal mechanical loads and longitudinal bone growth is to induce a bipedal gait or posture in an experimental animal model. Animal models are appropriate for testing the role of bipedalism on longitudinal bone growth because it is well established that bone is responsive to mechanical loads placed upon them across a wide variety of taxa. It should be noted that the degree of response may vary since the bony response to mechanical forces may scale differently in rodents than it does in humans [20]. However, because this study is looking at patterns of response, rather than correlating specific magnitudes of force to longitudinal growth outcomes, a rodent model presents a reasonable compromise as a model organism due to their relatively fast life history (see Methods).

One way to test the relationship between bipedal mechanical loads and longitudinal bone growth is to induce a bipedal gait or posture in an experimental animal model for fixed periods of time under specific loading conditions. Previous studies which have induced bipedal posture and gait in rodent models have relied upon forelimb amputation to unload the forelimbs (e.g.,[21–29]). These studies relied upon uncontrolled movements in a cage environment to load the hindlimbs. Moreover, one study found that rats with amputated forelimbs took on bipedal postures just as much as controls while in their cages [27]. Therefore, the impact of bipedal mechanical loads on longitudinal growth is still unclear and more study is required to test this relationship.

In this study, rats are used as a model to explore how mechanical loads from bipedal gait and posture modify longitudinal bone growth using five experimental groups which test a variety of loading conditions. Bipedal gait and posture is induced using a custom built apparatus mounted to a treadmill which controls the amount of joint loading experienced by the hindlimbs while keeping other variables relatively constant, including diet and environment. The amount of loading can be adjusted by modifying the amount of vertical force on the torso (see Methods). This experimental design tests three hypotheses that relate to how mechanical loads may alter longitudinal bone growth.

**H**_**1**_: Increasing the magnitude of hindlimb joint loads that result from a shift to bipedal locomotion will alter longitudinal growth and ultimately the length of the femur and the tibia
**H**_**2**_: A shift to bipedal locomotion while maintaining the magnitude of hindlimb joint loads normally experienced during quadrupedal locomotion will not alter longitudinal growth, and ultimately the length of the femur and tibia
**H**_**3**_: Increasing the magnitude of hindlimb joint loads that result from a shift to bipedal postural support will alter longitudinal growth or the length of the femur and the tibia

Previous work using this rat model, which tested how bipedal loading shapes axial anatomy, found significant differences in both absolute and percentage changes in vertebral wedging and percentage changes in sacral articular surface areas, consistent with expectations for adaptation to bipedal behaviors [30]. Therefore, this model has demonstrated utility for exploring how bipedal loading may influence other aspects of bone growth. Experimentally modeling the quadrupedal to bipedal transition in an animal model can provide important data and context for understanding the role of mechanical loading history on long bone development.

## Methods

Female Sprague-Dawley rats (*Rattus norvegicus*; Harlan, Indianapolis, IN, USA) were acquired at three weeks of age (the youngest age available from the vendor) and were allowed one week of acclimation. Rats were housed in a temperature and humidity controlled room using a 12-hour day-night light cycle. All rats were allowed *ad libitum* access to food and water and were group housed in cages (3 rats per cage) containing wood shaving bedding and a PVC tube. Cages were standard laboratory polycarbonate rat pans (19” x 10-1/2” x 8”) and were not equipped with an exercise wheel or any other method of enrichment. Exercise procedures, which occurred outside of the cage environment, were conducted during the light cycle.

Beginning at four weeks of age, rats were randomly assigned to each of the five experimental groups (n = 14/group): (1) “fully loaded” bipedal walking with nearly all body mass shifted to the hindlimbs (~90% of body mass; mechanical loading from locomotor forces), (2) bipedal walking with a shift to bipedalism but with a typical amount of body mass supported by the hindlimbs (45% body mass, the average amount supported by quadrupedal rat hindlimbs [31], (3) bipedal standing with nearly all body mass shifted to the hindlimbs (~90% body mass; mechanical loading from postural support), (4) quadrupedal walking (normal quadrupedal locomotor support), and (5) no exercise control (rats remain in cages). Here, “fully loaded” is ~90% of body mass because the harness system did not allow for the hindlimbs to support 100% of body mass. Because the treadmill was not instrumented with force plates, shifts to bipedal mechanical loads is an assumption based on animals walking with a bipedal gait or taking on a bipedal posture that was supported by the harness system (see description below). The quadrupedal and no exercise groups served as controls.

Rats engaged in their assigned behavior for a period of 12 weeks. Previous work demonstrates that in rats, longitudinal tibial growth is nearly exponential through 64 days of age, followed by a deceleration phase with no statistically significant growth occurring after 20 weeks [32]. In this study, rats engage in their assigned behaviors for approximately 5 weeks during periods of peak growth. The experiment also ends within the growth window of 20 weeks. Therefore, it can be assumed that these methods reliably captured the response in longitudinal long bone growth to bipedal mechanical loading regimes.

**Fig 1:**
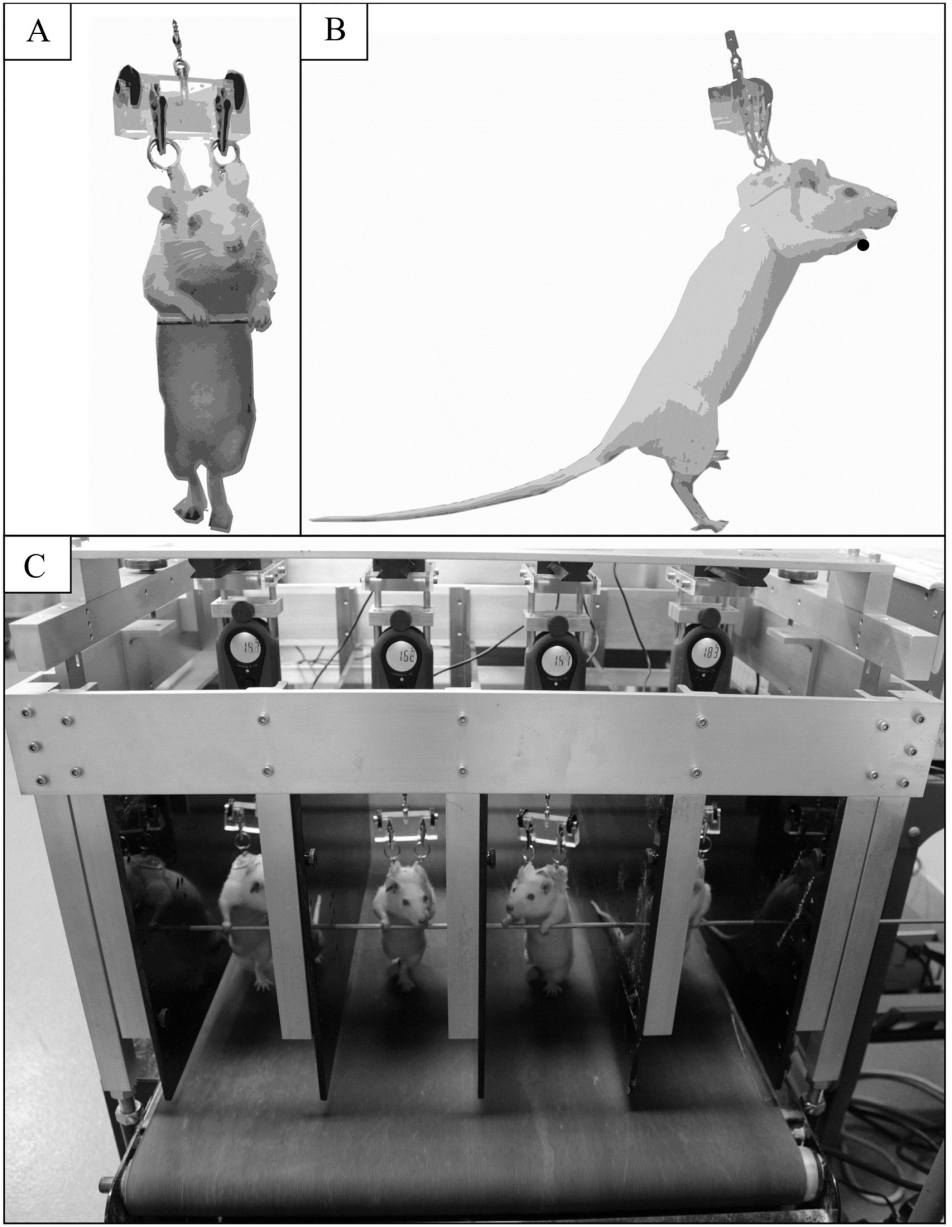
Harness system for bipedal walking and posture in rats. Rats walking bipedally in front view (A) and side view (B), using a harness system mounted on a large animal treadmill and a horizontal support bar, configured in a four lane configuration (C). A hanging scale was mounted above each rat that attaches to a jacket worn by each rat that measured the amount of upward force on the torso (as a percentage of body mass), which was recorded by a data logger over the course of each exercise bout during the 12-week experimental period. Reprinted from [33] under a CC BY license, with permission from Foster, original copyright 2017. Figure available at https://dx.doi.org/10.6084/m9.figshare.5459749.

Bipedal walking and standing in rats was accomplished through the use of a custom-built harness system mounted on a large animal treadmill (Jog A Dog LLC, Ottawa Lake, MI, USA) in a four lane configuration, (see Fig 1). Bipedal rats received postural support from the hanging scale, which is attached to their torso and helps maintain a vertical trunk orientation, and the use of a bar that runs horizontally across the trackway to hold on to with their forelimbs for stability. The vertical support of the torso is provided by two vertical wires attached near each shoulder to a rat jacket (SAI Infusion Technologies, Lake Villa, IL, USA), which are connected to a hanging scale (American Weigh, Cummings, GA, USA) in each lane above the rat to monitor load during the experiment. Each hanging scale is mounted to the harness frame such that height of each of the scales can be adjusted independently. This setup allows for adjustment of the amount of load applied to the hindlimbs of each rat at any time during the experimental period (i.e., increasing the height of the scale takes load off the hindlimbs, decreasing the height of the scale adds more load to the hindlimbs). In this study, any mass not recorded by the scale was assumed to be supported by the hindlimbs (i.e., if 10% of body mass is recorded on the scale, 90% is assumed to be supported by the hindlimbs). Each scale is also attached to a runner above each of the four lanes to accommodate fore-aft movement when on the treadmill.

Before daily exercise, each rat was weighed wearing the equipment and the position of the hanging scale was set to place the appropriate amount of load on the hindlimbs (e.g., 90% of body mass). Any mass taken off the hindlimbs and onto the forelimbs by pushing up or hanging off the bar at any point during daily exercise resulted in an adjustment in the height of the scale and thus an alteration of the amount of upward force on the torso. Each scale is connected to a data logger (MC-Measurement Computing, Norton, MA, USA) recording the voltage output at 2 Hz, to monitor and record hindlimb loading during each experimental period. This recording rate used for this experiment was the maximum recording rate possible for this data logger for a 60-minute recording. However, this recording rate resulted in 7200 data points per experimental period, per rat, which were used to calculate a mean percentage of body mass. These parameters provided an effective compromise given that each rat had recordings from 60 experimental periods.

Voltages for each scale, for each rat, for each day, were saved as *.csv files, labeled by scale number and animal ID. Scales were calibrated each day by placing four calibration weights of known mass on each scale to measure the voltage output. A least-squares linear calibration curve was fitted to these data to produce a formula to calculate the relationship between voltage and mass (grams). This calibration curve was used to calculate the mass recorded by each scale for each rat over every 60-minute exercise bout, over the 12-week experiment. These calculations were made using a custom script written in MATLAB 2014a (Mathworks, Natick, MA, USA).

Scale data was collected for the fully loaded bipedal, partially loaded bipedal, and standing groups. Rats were exposed to their assigned behaviors over the twelve-week experimental period, exercising five days a week, for a 60-minute bout. In the first week of exercise, rats underwent behavior training where they were exposed to increasing time intervals each day until they could engage in the behavior for the full 60-minute duration. Rats in the quadrupedal control group were exercised using a normal gait while wearing a rat jacket attached to the scales (with sufficient slack in the vertical lines) for the same period as other experimental groups. The harness system was not able to measure hindlimb loading from the quadrupedal group as this would require upward force on the torso, and thus decreased forelimb loading. Therefore, quadrupedal hindlimb loading was assumed to be typical (i.e., 45% of body weight [31].

The treadmill belt speed was set at the beginning of the experiment (Week 1) and was based on what was deemed to be a visually comfortable pace (there was no visible distress by the animals which was determined by erratic behavior or not keeping up with the treadmill belt speed) for the bipedal walking groups (~0.13 m/s). This speed was converted to a Froude number (0.038), V^2^(*Lg*)^-1^, where *V* is velocity, *L* is limb length, and *g* is gravitation acceleration, to ensure dynamic similarity across all experimental groups and throughout the twelve weeks of the experiment as their limbs grew longer [34]. Limb length (L) was measured as the effective hip height, which was measured from the greater trochanter to the treadmill belt prior to each experimental day using a measuring tape. This external measure of limb length was only used for Froude number calculations. Froude numbers were used to adjust the belt speed throughout the experimental period. The treadmill belt (and Froude number) was the same for all animals on each experimental day as their hindlimb lengths and hip heights were similar.

To track skeletal dimensions, *in vivo* μCT scans were taken before the start of the experiment (Week 0), and repeated at three week intervals throughout the experiment, such that there were five scans in total (Week 0, 3, 6, 9, and 12), using a small animal scanner (Inveon, Siemens Medical Solutions, Malvern, PA, USA). To take *in vivo* scans, rats were anesthetized using isoflurane (3% induction, 1.5% maintenance using 1.5L of O_2_ per minute). Scan parameters are shown in Table 1. CT scans were reconstructed using COBRA software (Exxim, Pleasanton, CA, USA.

**Table 1:** μCT scan parameters used in this study.

Parameter	Value
Voltage	80 kV
Current	500 μA
Exposures	440
Exposure Time	475 ms
Binning	Factor of 4
Gantry Rotation	220°
Voxel Size	105 μm

Amira (v. 5.4.3; FEI Visualization Sciences Group, Hillsboro, OR, USA) was used to generate isosurfaces which were manually segmented, in order to take 3D distance measurements of femur and tibia lengths using the 3D ruler. Distance measurements for the femur and the tibia are an average of three measurements for each bone. Definitions for measurements are shown in Table 2. Relative changes in long bone lengths, used as a size correction in this study, were calculated using the percentage difference between measurements taken at the first scan prior to the initiation of the experiment (i.e., Week 0) and each subsequent scan (i.e., Week 3, 6, 9, and 12). This measure tracks relative changes in length compared to the length at the start of the experiment (e.g., Week 12 –Week 0/Week 0). Differences from baseline value (i.e., Week 12 –Week 0) are also compared to measure absolute changes in bone length.

**Table 2:** Definitions for length measurements for the femur and tibia.

Measurement	Definition
Femur Length	The maximum distance from the most proximal point at the greater trochanter to the most distal point on the lateral condyle
Tibia Length	The most anterosuperior point on the tibial plane to the most distal point on the medial epicondyle

The fully loaded bipedal walking, partially loaded bipedal walking, and standing quadrupedal groups had their body masses measured with a digital scale prior to each experimental day to calculate the amount of body weight that needed to be offset by the hanging scales. Body masses were measured for all rats (including the quadrupedal and no activity controls) every third week, corresponding to the day of each μCT scan. At the end of the 12-week experiment, animals were sacrificed by CO_2_ overdose. All methods and procedures used in this study were approved by the University of Arizona IACUC (Protocol #10–164). The methods and data reported in this study follow ARRIVE guidelines [35].

All statistical analyses for this study were conducted in R [36]. The hindlimb loading data from the scales were first analyzed using an ANOVA with the experimental group as the categorical factor to test for significant differences (at p<0.05). If significant differences were found, a Fisher’s least significant difference (LSD) analysis was used to control for multiple comparisons (using the agricolae package) [37]. Segment length data were first analyzed using a mixed-effect model (using the lme function) [38]. The random factor for all mixed-effect analyses was the individual animal. A mixed-effect model is most appropriate for testing the hypotheses in this study as it allows for adjustment of the degrees of freedom to account for variation among individuals and error terms to account for repeated measures of the same individual. Results from the mixed-effect model and interaction terms were first analyzed using an ANOVA, which if significant, was followed by post-hoc analyses using least-squares mean contrasts (using the lsmeans function), with p-values adjusted for multiple comparisons (using the ‘fdr’ method) [39]. Statistical analyses were also supplemented using the data.table [40], reshape2 [41], and tidyverse packages [42]. Figures were made using ggplot2 [43] and cowplot [44].

## Results

**Fig 2:**
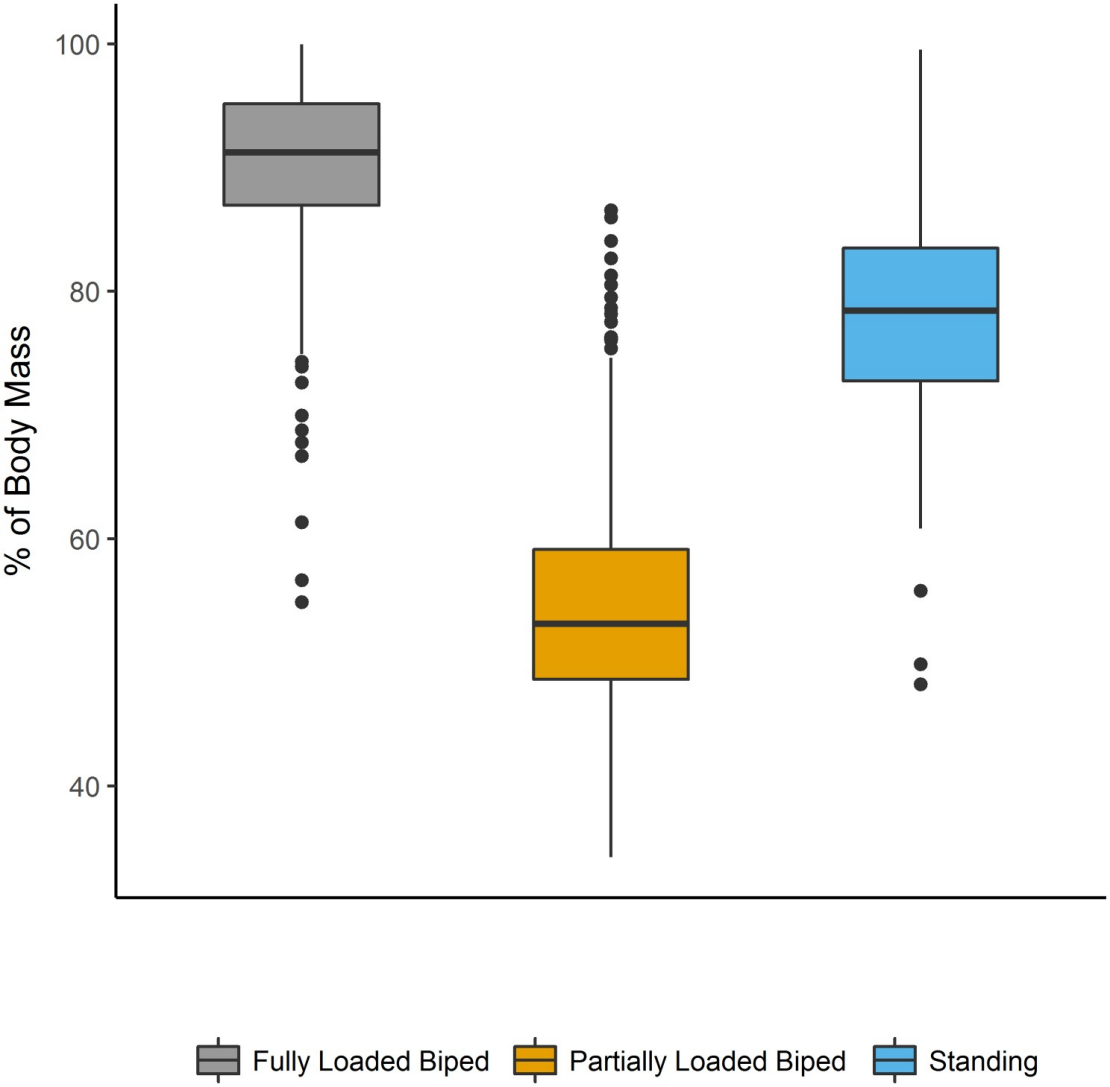
Mean hindlimb loading for each experimental group. Boxplot of the mean amount of load experienced by the hindlimbs (as a percentage of body mass) in each experimental group. The horizontal lines indicate the median (of the daily loading means), the outer boundaries of the box represent the interquartile range, the whiskers indicate the minimum and maximum, respectively, and the individual points represent outliers. Data are from each 60-minute period over the twelve week experiment. All comparisons are significantly different (p<0.001). Reprinted from Foster [45] under a CC BY license, with permission from Foster, original copyright 2018. Figure available at https://doi.org/10.6084/m9.figshare.5462065.v4 under a CC-BY 4.0 license.

There were significant differences among the experimental groups for the amount of hindlimb loading, calculated as the mean percentage of body mass experienced by the hindlimbs for each rat for each 60 minute experimental period, over the 12-week experiment (F_[41,2]_ = 110.1, p<0.001). A follow-up Fisher’s LSD post-hoc test found significant differences in pairwise comparisons between all three groups (p<0.001; Fig 2). The mean loading amount experienced by the fully loaded bipedal group was 90.2% (±7.2% [SD]) of body mass, the partially loaded group experienced 54.5% (± 8.9% [SD]) of body mass, and the standing group experienced 78.5% (± 8.2% [SD]) of body mass. There were no measured hindlimb loads for the quadrupedal control group because loading was measured via a hanging scale, which measures the amount of body mass offset by a vertical force on the torso (see Methods).

The targeted hindlimb loading values for each experimental group were within one standard deviation of the mean of the mean values measured by the scales. However, there is some variation within each experimental group, including mean values that overlap with other groups (see Fig 2). This result can be attributed to a study design which tests the impact of mechanical loading on longitudinal bone growth from gait and posture, which results in fluctuating loads over each step. Therefore, while there are outliers for mean values for daily exercise bouts, the majority of daily loading percentages for the fully loaded (90% of body mass) and the partially loaded (45% of body mass) bipedal groups were close to the target mechanical loads.

The rats in the standing group required constant monitoring to ensure they were loading their limbs properly, rather than taking on a more compliant (flexed joints) position. If rats adjusted their posture by taking on a flexed position, the presence of a gloved hand corrected the behavior. Therefore, while the standing group loaded their limbs more than the partially loaded group, the rats in the standing group experienced reduced hindlimb loading relative to the fully loaded bipedal group.

Body masses measured at three-week intervals throughout the 12-week experiment were compared between groups using a linear mixed-effect model. A pair-wise group comparison (p-values controlled for multiple comparisons using the FDR method) found no significant differences between the experimental groups (F_[4,68]_ = 1.535, p = 0.202). Group means and standard deviations for body mass are shown in S1 Table. A boxplot of body masses by group, for each three-week interval is located in S1 Fig.

**Fig 3:**
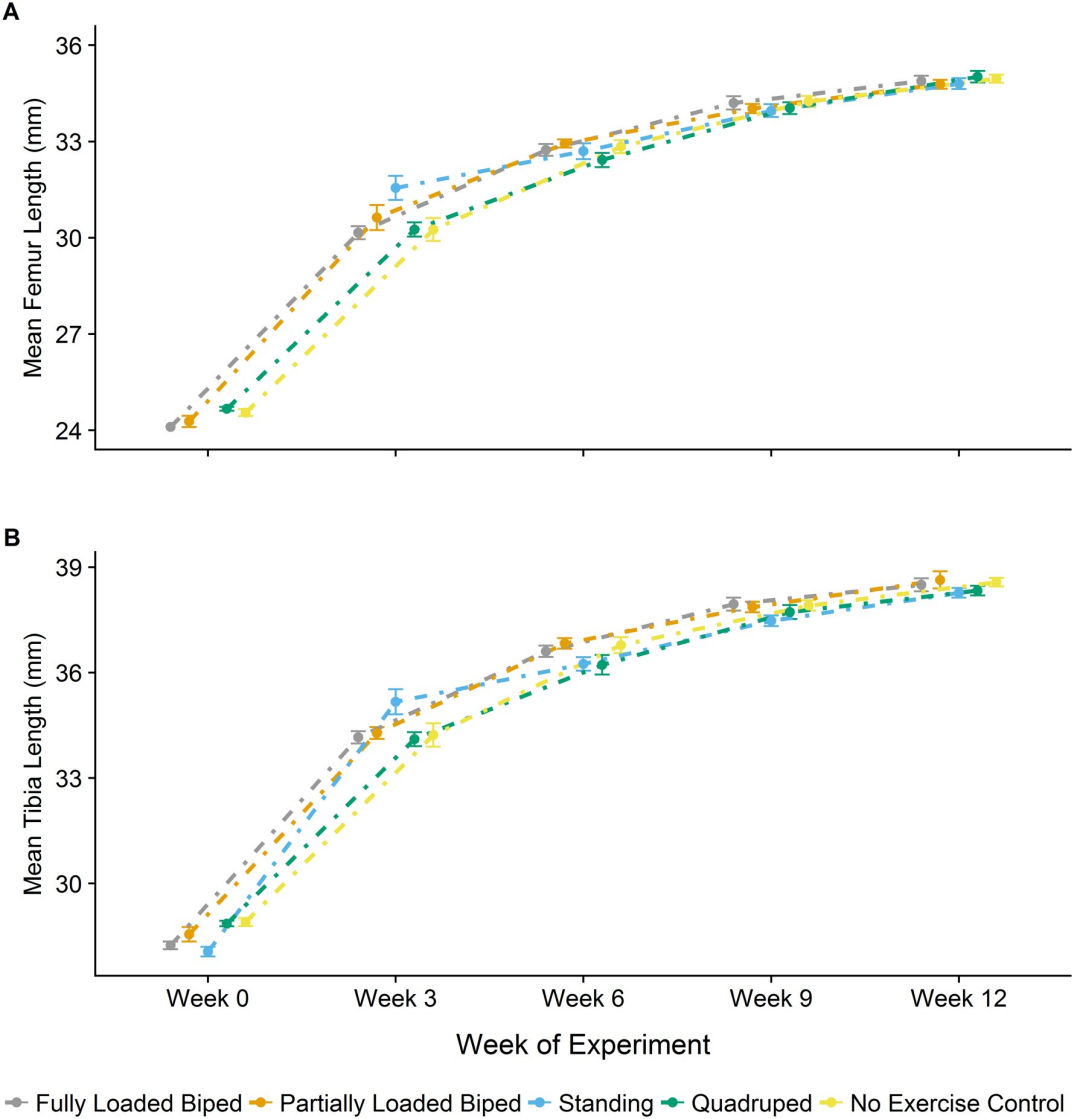
Mean lengths of the femur and tibia for each experimental group over the 12-week experiment. Line plots for the femur (A) and tibia (B) for each experimental group that were measured from each μCT scan, every third week, over the 12-week experiment. The points represent the mean and the error bars represent the mean squared error for each group.

A mixed-effect model for the absolute lengths of the femur and tibia over the 12-week experiment were not significantly different between each of the experimental groups (Femur: F_[4,65]_ = 0.127, p = 0.906; Tibia: F_[4,65]_ = 0.319, p = 0.865). Mean hindlimb segment length values and standard deviations measured from each μCT scan (every third week) are shown in Table 3. Group means for each μCT scan for femur and tibia length over the 12-week experiment are also displayed in a line plot in Fig 3. The line plots show that all bipedal groups began the experiment with shorter hindlimb segment lengths, though differences in length were small, ranging from 2–4%. At the end of the experiment, bipedal group means were closer to the quadrupedal and no exercise group means.

**Table 3:** Mean femur and tibia lengths by group.

		Week 0		Week 3		Week 6		Week 9		Week 12	
Experimental Group	Segment	*Mean (mm)*	*SD*	*Mean (mm)*	*SD*	*Mean (mm)*	*SD*	*Mean (mm)*	*SD*	*Mean (mm)*	*SD*
Fully Loaded Biped	Femur	24.11	0.48	30.15	0.76	32.74	0.68	34.20	0.77	34.88	0.61
Partially Loaded Biped	Femur	24.28	0.66	30.63	1.46	32.93	0.48	34.03	0.52	34.78	0.52
Standing	Femur	23.98	0.54	31.55	1.41	32.70	0.93	33.96	0.74	34.80	0.64
Quadruped	Femur	24.67	0.22	30.26	0.83	32.42	0.82	34.04	0.69	35.02	0.68
No Exercise Control	Femur	24.55	0.41	30.26	1.34	32.84	0.76	34.25	0.65	34.96	0.47
Fully Loaded Biped	Tibia	28.24	0.41	34.16	0.66	36.60	0.60	37.95	0.69	38.50	0.69
Partially Loaded Biped	Tibia	28.55	0.77	34.28	0.62	36.83	0.56	37.86	0.57	38.64	0.91
Standing	Tibia	28.06	0.52	35.17	1.33	36.25	0.72	37.47	0.56	38.27	0.51
Quadruped	Tibia	28.86	0.30	34.11	0.73	36.22	1.05	37.72	0.74	38.33	0.51
No Exercise Control	Tibia	28.90	0.41	34.22	1.25	36.78	0.85	37.91	0.56	38.57	0.46

Mean lengths of the femur and tibia and standard deviations (SD) by group, measured every third week from each μCT scan.

**Fig 4:**
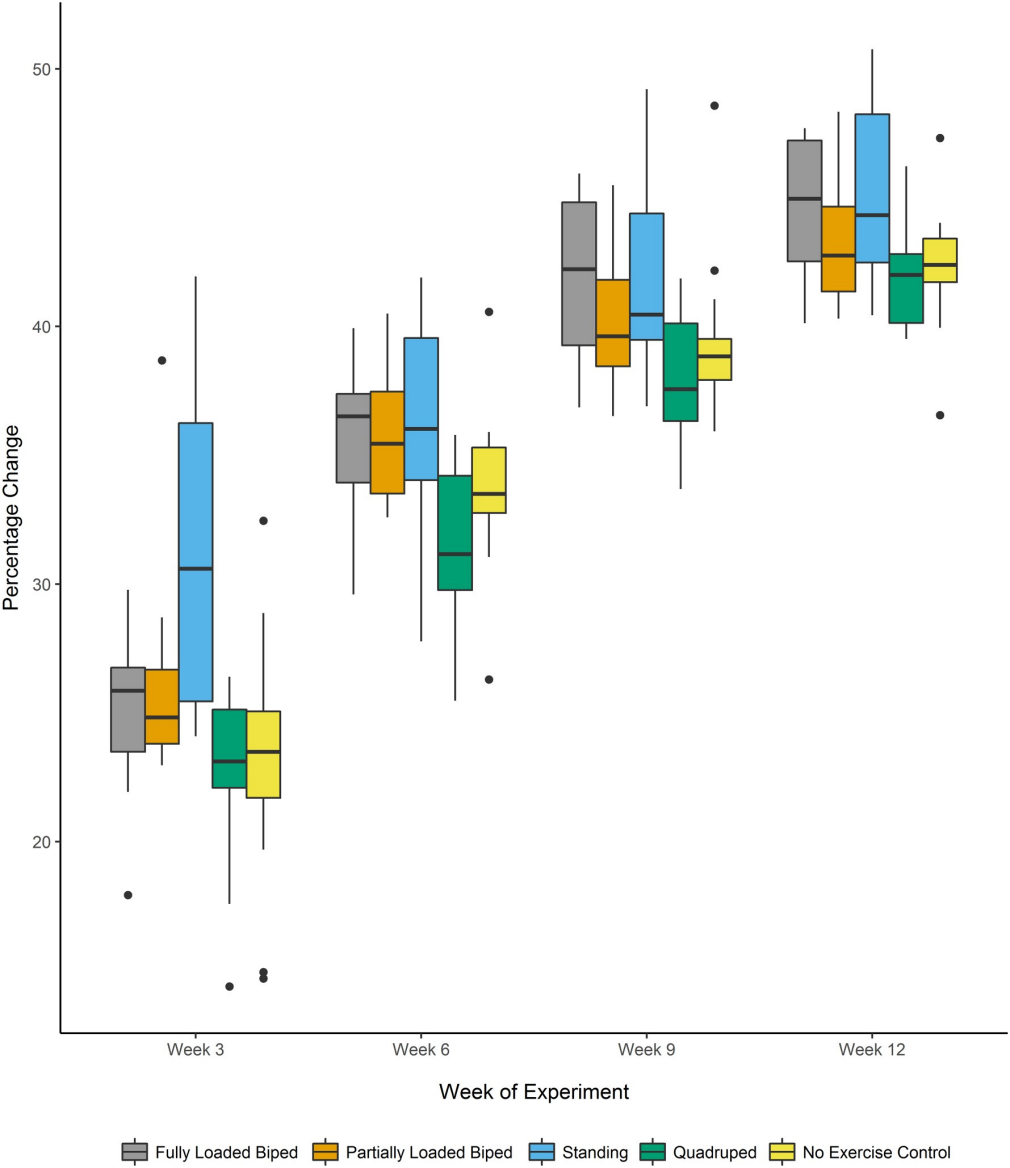
Percentage change in femur length over the 12-week experiment. Boxplot of the percentage change in femur length at weeks 3, 6, 9, and 12 relative to the length at the beginning of the experiment. The horizontal lines indicate the median, the outer boundaries of the box represent the interquartile range, the whiskers indicate the minimum and maximum, respectively, and the individual points represent outliers.

**Fig 5:**
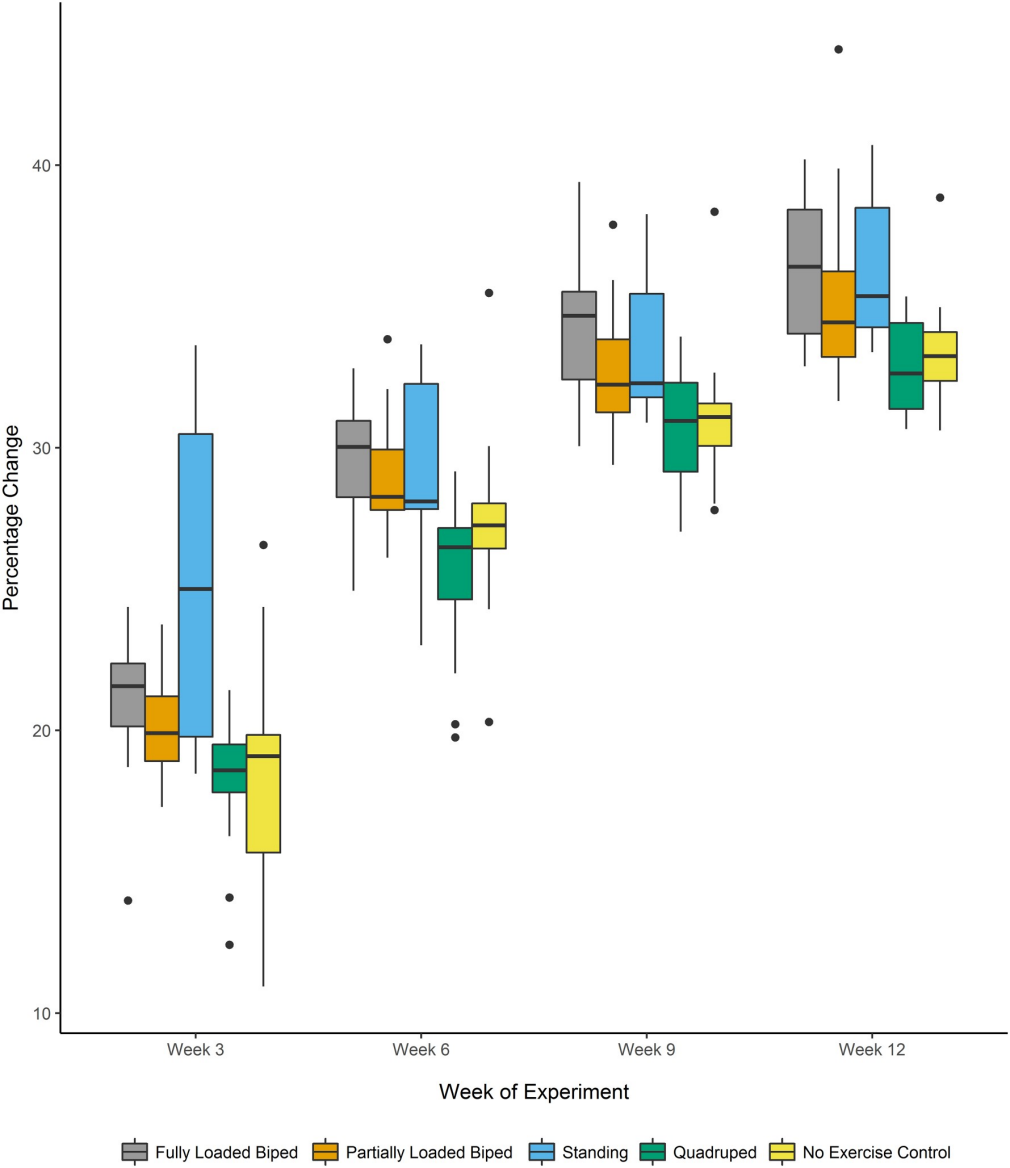
Percentage change in tibia length over the 12-week experiment. Boxplot of the percentage change in tibia length at weeks 3, 6, 9, and 12 relative to the length at the beginning of the experiment. The horizontal lines indicate the median, the outer boundaries of the box represent the interquartile range, the whiskers indicate the minimum and maximum, respectively, and the individual points represent outliers.

Results from the mixed-effect model show significant differences in the relative changes in length (percentage change) over the course of the 12 week experiment for the femur and tibia between the experimental groups (Femur: F_[4,65]_: 15.501, p<0.001; Tibia: F_[4,65]_: 13.929, p<0.001). Mean values for the percentage changes in the length of the femur and tibia are located in Table 4. A multiple comparisons post-hoc analysis found that the fully loaded bipedal walking and the standing groups, which experienced mechanical loads associated with a shift to bipedal locomotion and posture, had significantly greater percentage changes in length in the femur and tibia when compared to the quadrupedal and the no exercise control groups. The effect size for percentage change from Week 12 to Week 0 was large. The partially loaded bipedal group showed significantly different percentage change in length for all segments when compared to the quadrupedal group with a large effect size, but there were no significant differences when compared to the no exercise control group. Results from the least-squares means post-hoc analyses from the linear mixed-effect model are shown in Table 5. Boxplots demonstrating percentage changes in limb segment length by group are located in Figs 4 and 5.

**Table 4:** Mean percentage differences in femur and tibia lengths by group.

		Week 3—Week 0		Week 6—Week 0		Week 9—Week 0		Week 12—Week 0	
Experimental Groups	Segment	*Mean*	*SD*	*Mean*	*SD*	*Mean*	*SD*	*Mean*	*SD*
Fully Loaded Biped	Femur	25.09	3.02	35.81	2.69	41.89	3.02	44.71	2.69
Partially Loaded Biped	Femur	26.15	4.05	35.72	2.60	40.22	2.49	43.34	2.46
Standing	Femur	31.63	6.68	36.35	3.83	41.66	3.69	45.17	3.48
Quadruped	Femur	22.64	3.36	31.42	3.13	37.97	2.48	41.92	1.97
No Exercise Control	Femur	23.22	4.74	33.77	3.11	39.51	3.03	42.40	2.36
Fully Loaded Biped	Tibia	20.97	2.51	29.64	2.28	34.40	2.74	36.35	2.46
Partially Loaded Biped	Tibia	20.09	1.68	29.04	2.10	32.67	2.34	35.37	3.40
Standing	Tibia	25.40	5.93	29.21	3.13	33.58	2.67	36.43	2.69
Quadruped	Tibia	18.19	2.52	25.51	3.03	30.72	2.17	32.84	1.64
No Exercise Control	Tibia	18.43	4.30	27.29	3.29	31.20	2.50	33.49	1.90

The mean percentage change in length [e.g., ((Week3-Week0)/Week0)] of the femur and tibia and standard deviation (SD) for each group.

**Table 5:** Least-squares means multiple comparisons table for percentage change in femur and tibia length.

Contrasts	Segment	Estimate	SE	df	t.ratio	Adjusted p-value	Effect Size
Fully Loaded Biped—Partially Loaded Biped	Femur	0.52	1.02	65	0.51	0.612	0.534
Fully Loaded Biped—Standing	Femur	-1.83	1.02	65	-1.80	0.110	-0.146
Fully Loaded Biped—Quadruped	Femur	3.38	1.02	65	3.33	**0.005**	**1.184**
Fully Loaded Biped—No Exercise Control	Femur	2.15	1.02	65	2.11	**0.064**	**0.914**
Partially Loaded Biped—Standing	Femur	-2.35	1.02	65	-2.31	**0.049**	-0.608
Partially Loaded Biped—Quadruped	Femur	2.86	1.02	65	2.82	**0.016**	0.636
Partially Loaded Biped—No Exercise Control	Femur	1.63	1.02	65	1.60	0.142	0.389
Standing—Quadruped	Femur	5.21	1.02	65	5.13	**<.0001**	**1.149**
Standing—No Exercise Control	Femur	3.98	1.02	65	3.91	**0.001**	**0.932**
Quadruped—No Exercise Control	Femur	-1.23	1.02	65	-1.22	0.254	-0.219
Fully Loaded Biped—Partially Loaded Biped	Tibia	1.04	0.91	65	1.15	0.318	0.328
Fully Loaded Biped—Standing	Tibia	-0.82	0.91	65	-0.90	0.388	-0.032
Fully Loaded Biped—Quadruped	Tibia	3.52	0.91	65	3.88	**0.001**	**1.678**
Fully Loaded Biped—No Exercise Control	Tibia	2.73	0.91	65	3.01	**0.009**	**1.298**
Partially Loaded Biped—Standing	Tibia	-1.86	0.91	65	-2.05	0.074	-0.344
Partially Loaded Biped—Quadruped	Tibia	2.48	0.91	65	2.73	**0.016**	**0.950**
Partially Loaded Biped—No Exercise Control	Tibia	1.69	0.91	65	1.86	0.097	0.683
Standing—Quadruped	Tibia	4.34	0.91	65	4.78	**0.000**	**1.611**
Standing—No Exercise Control	Tibia	3.55	0.91	65	3.91	**0.001**	**1.260**
Quadruped—No Exercise Control	Tibia	-0.79	0.91	65	-0.87	0.388	-0.368

Results from the mixed-effect model comparing the mean percentage change [e.g., (Week12-Week0)/Week0))] in the length of the femur and tibia, including the standard error (SE), degrees of freedom (df), t ratio (t.ratio), and p-value for each group comparison. Bolded values indicate significant differences. Effect sizes (Cohen’s D) are calculated from the percentage change in the femur and the tibia from the beginning and the end of the experiment [i.e., ((Week12-Week0)/Week0)]. Bolded values indicate large effect sizes (≥0.8).

**Fig 6:**
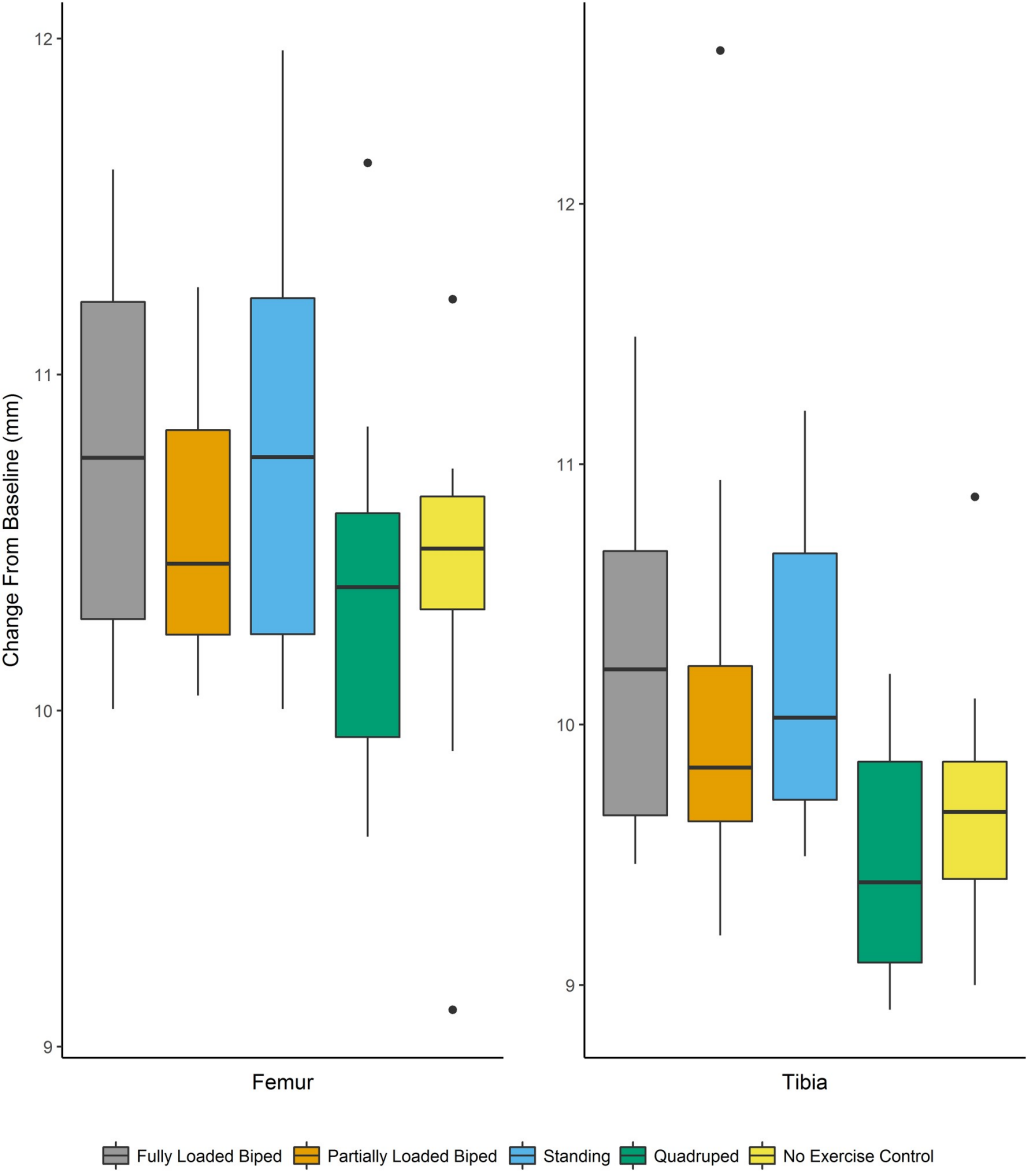
Change from baseline in the femur and tibia over the 12-week experiment. Boxplot of the change in length from baseline (i.e., Week 12—Week 0) in the femur and tibia. The horizontal lines indicate the median, the outer boundaries of the box represent the interquartile range, the whiskers indicate the minimum and maximum, respectively, and the individual points represent outliers.

Comparisons were also made using the absolute change from baseline (i.e., Week 12-Week 0) using a mixed-effect model with body mass as a fixed effect (covariate) to control for body size. This analysis showed significant differences across experimental groups for change in the length of the femur (F_[4,64]_: 2.945, p = 0.027) and tibia (F_[4,64]_: 4.864, p = 0.002). A multiple comparisons post-hoc analysis found significant differences in the change in femur length between the standing and no exercise control groups. For the tibia, significant differences were found between all bipedal groups and both the quadrupedal and no exercise control groups (control groups). Results for the change in baseline mixed-effect model are located in Table 6 and Fig 6.

**Table 6:** Changes from baseline (Week 12 –Week 0) for the femur and tibia.

Segment	Estimate	SE	df	t.ratio	Adjusted p-value	Effect Size
Femur	0.085	0.188	64	0.449	0.655	0.564
Femur	-0.208	0.187	64	-1.109	0.388	-0.077
Femur	0.174	0.194	64	0.898	0.466	0.790
Femur	0.402	0.183	64	2.198	0.141	0.712
Femur	-0.292	0.183	64	-1.599	0.229	-0.569
Femur	0.089	0.184	64	0.487	0.655	0.357
Femur	0.317	0.191	64	1.664	0.229	0.242
Femur	0.382	0.184	64	2.072	0.141	0.777
Femur	0.610	0.189	64	3.222	**0.020**	0.704
Femur	0.228	0.197	64	1.157	0.388	-0.118
Tibia	-0.010	0.225	64	-0.046	0.963	0.233
Tibia	-0.116	0.224	64	-0.517	0.765	0.076
Tibia	0.525	0.232	64	2.264	**0.045**	**1.400**
Tibia	0.620	0.219	64	2.834	**0.019**	**1.036**
Tibia	-0.105	0.219	64	-0.482	0.765	-0.172
Tibia	0.535	0.220	64	2.438	**0.035**	**0.908**
Tibia	0.630	0.228	64	2.764	**0.019**	0.610
Tibia	0.641	0.220	64	2.907	**0.019**	**1.354**
Tibia	0.736	0.226	64	3.251	**0.018**	**0.980**
Tibia	0.095	0.236	64	0.403	0.765	-0.437

Results from the mixed-effect model comparing the mean change from baseline (i.e., Week 12 –Week 0) in the length of the femur and tibia while controlling for body mass, including the standard error (SE), degrees of freedom (df), t ratio (t.ratio), and p-value for each group comparison. Bolded values indicate significant differences. Effect sizes (Cohen’s D) are calculated from the change in baseline of the femur and the tibia from the beginning and the end of the experiment [i.e., (Week12-Week0)]. Bolded values indicate large effect sizes (≥0.8).

Overall, the results from this study offer partial support for the first and third hypotheses and conditional support for the second. The first and third hypotheses test whether joint loads from a shift of nearly all body weight to the hindlimbs moderate longitudinal growth for bipedal walking (H_1_) and standing (H_3_). These results offer partial support for both of these hypotheses because while there were no significant differences in absolute length, there were significant differences in measures of percentage change and change from baseline, which may suggest a change in growth *velocity*. However, results did vary depending on the metric used for comparison. Only the percentage change comparison showed significant differences in femur length when comparing both the control groups and the fully loaded bipedal and standing groups. The change from baseline comparison for tibia length showed significant differences between the control groups and both the fully loaded bipedal walking and standing groups (see Table 5).

The second hypothesis tests whether shifting to bipedal locomotion without altering the magnitude of joint loads will modify longitudinal bone growth. There were no significant differences in measures of percentage change in femur or tibia length between the partially loaded bipedal and the no activity control group (though there were significant differences between this group and the quadrupedal group [see Table 5]). Additionally, there were significant differences in the change from baseline measure for tibia length when comparing the partially loaded walking group and both the control groups (see Table 6). However, one mitigating circumstance is that the partially loaded group experienced average joint loads of 54.5% of body mass (the typical amount of joint loads on quadrupedal hindlimbs is ~45% [31]). Therefore, although the measured average value was similar, an argument can be made that the second hypothesis wasn’t completely tested.

## Discussion

The purpose of this study was to explore how bipedal locomotor and postural mechanical loads may moderate longitudinal bone growth in an animal model. This study used five different experimental groups to test the independent effects of a postural and locomotor shift to bipedal behaviors and the dose-dependent effects of force magnitude. While the study design cannot characterize the mechanical forces applied to bones (i.e., tension, compression, shear, etc.), it does offer insight into how bone growth is impacted by the average loading amounts experienced by the hindlimbs. The mechanical loading applied to animal hindlimbs was consistent (as measured by average values across all experimental days) and occurred during crucial growth periods of the tibia in the rat [32]. Ultimately, there were no absolute differences in length for the femur and tibia. However, there were significant differences in the percentage change in length for the fully loaded bipedal walking and bipedal standing groups when compared to both control groups, which appears to start a process of leveling off from weeks 9 to 12. This result is consistent with previous work which suggests that longitudinal bone growth in rat tibiae undergo rapid, logarithmic growth through 64 days of age. After this point, growth begins to slow with no detected growth after 20 weeks [32]. At weeks 9 to 12 of the experiment, the rats in this study are approximately 84 to 105 days of age (rats were 4 weeks of age [28 days] at the start of the experiment), which is consistent with growth rates in Horton et al. [32]. Because there were no differences in absolute values, but significant differences seen in measures of relative change, these bipedal mechanical loads may be altering growth velocity. However, the final bone length is still strongly influenced by genetics.

When comparing the change in length from baseline (while controlling for body mass), there were some important differences from the percentage change analyses. For femoral length, the only significant differences were seen between the standing and quadrupedal groups. For tibia length, all bipedal groups (fully loaded bipedal walking, partially loaded bipedal walking, and standing) showed significant differences with large effect sizes from the quadrupedal and no exercise control groups. This metric only uses the beginning and end points of the experiment and does not provide as much insight into growth rate (and velocity) from each three week interval that is apparent from the percentage change variables (see Figs 4 and 5). However, this metric does suggest that when taking body mass into account, a shift to bipedal walking with only a very small difference in hindlimb loading is sufficient to generate changes in longitudinal growth. Additionally, comparisons using change from baseline have a higher statistical power than looking at percentage change [46].

Parsing these results suggests that the magnitude of joint loads plays a role in moderating bone growth, and that in the case of bipedal loading, that threshold is reached at least at an average of 54.5% of body weight. Assuming that the quadrupedal group experienced hindlimb joint loads at or near 45% [31], this is a relatively small difference. However, these differences in growth are primarily seen in the tibia when controlling for body mass, which may be explained by differences in how the tibia was loaded relative to the femur or in the way that forces are transmitted (e.g., tensile, compressive, etc.) to the bone. Future work should explore how these bipedal behaviors translate to differences in loading using a combination of force plates, kinematic analysis, and strain gauges.

Future work should also continue to explore how growth plate dynamics are altered by abnormal conditions. One way to test whether bipedal mechanical joint loads may be altering growth velocity would be to conduct histomorphological analyses of the growth plate in cross-sectional samples using this animal model. Results which show greater numbers of hypertrophic chondrocytes and/or growth plate dimensions would suggest changes in growth velocity that are correlated to bipedal mechanical loads. Additionally, finite element analysis modeling would be advantageous for clarifying how loads that are not applied through external devices may be linked with alterations in longitudinal growth.

Overall, the results of this study suggest that the impact of mechanical loads on longitudinal bone growth are complex. Previous work has demonstrated that compressive loads suppress longitudinal growth, while lateral forces applied to epiphyses promote longitudinal growth [6,7,10,11]. In this study, loads applied to the hindlimbs were varied and not precisely applied from an external device. This suggests that linking specific behaviors with longitudinal growth may be difficult and poses challenges for experimental design. Previous work comparing exercised versus non-exercised pigs found differences in longitudinal growth which suggests that mechanical loading may explain some intraspecific variation in limb dimensions [9]. However, in this same study, the authors found that mechanical loading, bone length, and growth plate thickness are not necessarily linked. Another study found that exercised rats had no differences in femoral length when compared to non-exercised controls [47]. The conflicting results from different taxa suggest that more work is required to sort out how mechanical loads influences longitudinal bone growth and why organisms may have a different response to mechanical loads.

### Limitations of the study

There are limitations to this study which should be noted to contextualize these results. Primarily, the study design and equipment used to induce bipedal behaviors precluded the use of force plates, which would have provided more direct measures of how different mechanical loading regimes transmit force to the hindlimbs. These data would also be informative for calculating the number of loading cycles on the hindlimbs, which could potentially explain variation in change within experimental groups. There are also limitations imposed by the harness system, which induces a bipedal gait on a rat model that is accomplished with vertical support from a harness and a horizontal support from a cross bar. Although these rats were not able to accomplish bipedal locomotion and posture in a similar manner to other organisms like primates (that is, voluntarily for extended periods of time), this model still accomplishes the intended goal, which is to test how mechanical loads from bipedal behaviors impact longitudinal bone growth. Moreover, these constraints are assumed to be similar across individuals and represents an acceptable method for testing patterns in how mechanical loads from bipedal behaviors impacts the quadrupedal skeleton.

One additional limitation for interpreting the results of this study is that differences in the change in length are fairly small (~2–4%). This suggests that morphological changes mediated by mechanical loading have limitations which are constrained by the norms of reaction of bone (the range of phenotypic outcomes possible given environmental input). One additional possible explanation is that these results are driven in whole or in part by stochastic effects from catch-up growth. All bipedal groups had absolutely smaller tibiae at the beginning of the experiment than the quadrupedal and no activity control groups. Therefore, it is possible that differences in growth velocity could be driven by processes related to catch-up growth. However, it is difficult to distinguish catch-up growth from changes in growth velocity mediated by mechanical loading from bipedal behaviors.

### Conclusions

Five experimental groups of rats were used to explore the impact of mechanical loads from bipedal behaviors on longitudinal bone growth. These groups tested the independent effects of bipedal locomotion, posture, and the dose-dependent effects of force magnitude. The results demonstrate that while the absolute length values for limb length were not significantly different, there were significant differences for both percentage change and change from baseline, which suggests that mechanical loading from bipedal walking and posture, even with a very small increase in mechanical loads (relative to typical quadrupedal hindlimb loads) may alter longitudinal growth velocity in the femur and the tibia.

## Supporting information

S1 Fig Rat body masses over the 12-week experiment. A boxplot of body mass for each experimental group of rats, taken every three weeks, over the 12 week experiment. Reprinted from Foster [48] under a CC BY license, with permission from Foster, original copyright 2018. Figure available at https://doi.org/10.6084/m9.figshare.5910022.v3.
(TIFF)

S1 Table Body masses over the 12-week experiment. Mean body mass and standard deviation (SD) in grams for each group, measured prior to each μCT scan, at week 0 (before the experiment started), 3, 6, 9, and 12.
(DOCX)
