# Supplementary figures and images for: The impact of bipedal mechanical loading history on longitudinal long bone growth
Source: PLoS One. 2019 Feb 7;14(2):e0211692. doi: 10.1371/journal.pone.0211692 (PMC6366785; doi:10.1371/journal.pone.0211692)

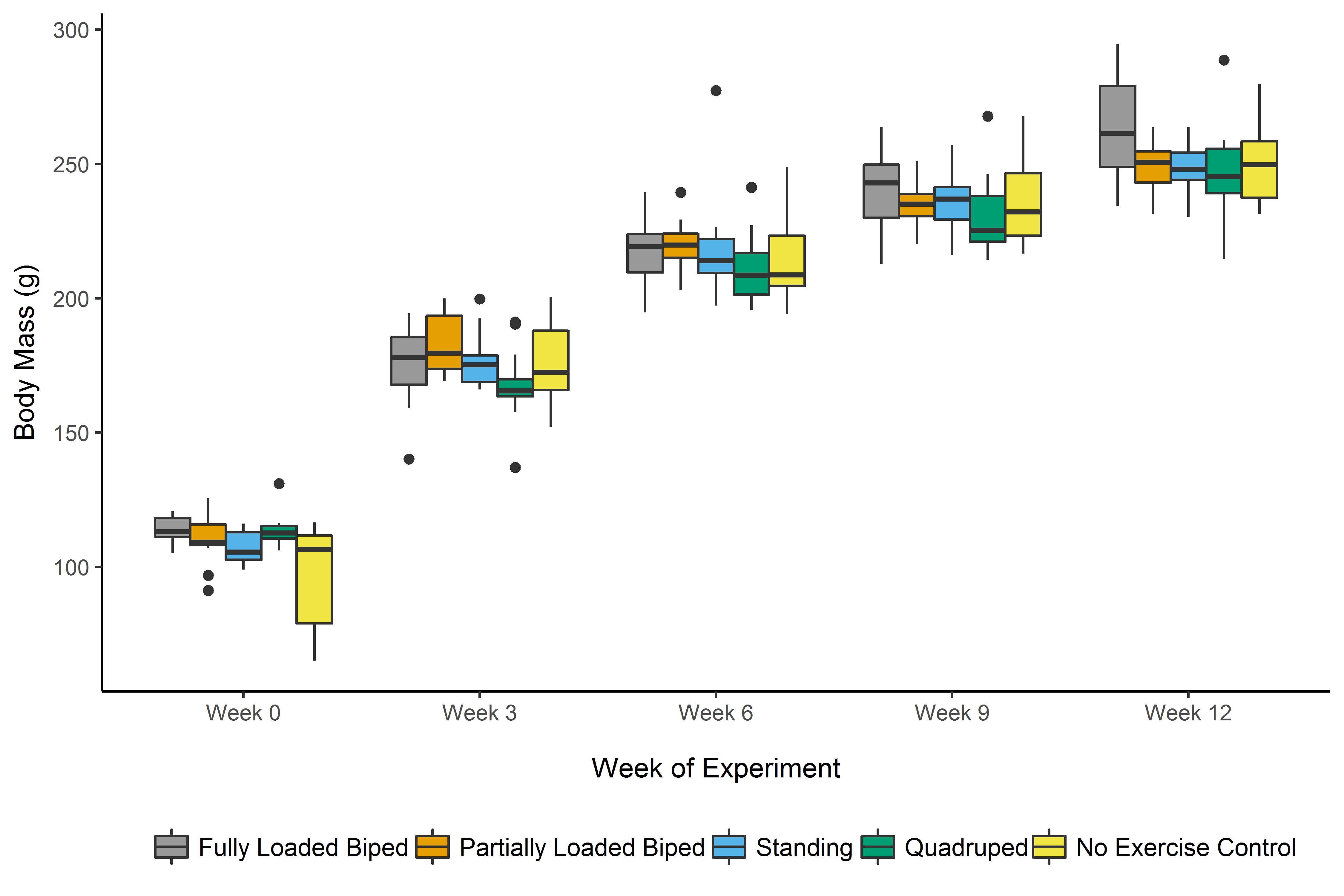

Supplement: S1 Fig — A boxplot of body mass for each experimental group of rats, taken every three weeks, over the 12 week experiment. Reprinted from Foster [48] under a CC BY license, with permission from Foster, original copyright 2018. Figure available at https://doi.org/10.6084/m9.figshare.5910022.v3. (TIFF) [file pone.0211692.s001.tiff]
