# Supplementary material for: The impact of bipedal mechanical loading history on longitudinal long bone growth
Source: PLoS One. 2019 Feb 7;14(2):e0211692. doi: 10.1371/journal.pone.0211692 (PMC6366785; doi:10.1371/journal.pone.0211692)
Supplement: S1 Table — Mean body mass and standard deviation (SD) in grams for each group, measured prior to each μCT scan, at week 0 (before the experiment started), 3, 6, 9, and 12. (DOCX) [file pone.0211692.s002.docx]

**S1 Table. Body Masses Over the 12-Week Experiment.**

|  | **Week 0** | | **Week 3** | | **Week 6** | | **Week 9** | | **Week 12** | |
| --- | --- | --- | --- | --- | --- | --- | --- | --- | --- | --- |
| **Experimental Groups** | *Mean (g)* | *SD* | *Mean (g)* | *SD* | *Mean (g)* | *SD* | *Mean (g)* | *SD* | *Mean (g)* | *SD* |
| Fully Loaded Biped | 113.71 | 4.87 | 175.54 | 13.86 | 217.45 | 12.91 | 240.67 | 15.68 | 263.54 | 20.04 |
| Partially Loaded Biped | 110.35 | 8.71 | 182.16 | 10.84 | 217.61 | 7.44 | 235.34 | 9.09 | 249.14 | 9.37 |
| Standing | 107.42 | 5.89 | 177.71 | 10.20 | 218.24 | 18.87 | 235.47 | 10.73 | 247.61 | 9.23 |
| Quadruped | 113.59 | 5.81 | 167.45 | 13.42 | 211.31 | 12.57 | 230.91 | 14.65 | 247.43 | 16.64 |
| No Exercise Control | 97.64 | 19.94 | 175.04 | 15.06 | 213.09 | 15.63 | 234.84 | 14.84 | 250.75 | 16.04 |

Mean body mass and standard deviation (SD) in grams for each group, measured prior to each µCT scan, at week 0 (before the experiment started), 3, 6, 9, and 12.
